# Orofacial Pain Symptoms Among Chinese Older Adults in the Last Year of Life

**DOI:** 10.1093/geroni/igab046.115

**Published:** 2021-12-17

**Authors:** Yaolin Pei, Xiang Qi, Xi Chen, Bei Wu

**Affiliations:** 1 New York University, New York, New York, United States; 2 Rory Meyers College of nursing, New York, New York, United States; 3 University of Iowa, Iowa City, Iowa, United States

## Abstract

The aims of this study were to examine the prevalence of orofacial pain symptoms and its associated factors in Chinese older adults in the last year of life. We retrospectively followed 1,646 participants (60 years or older) in the last year of life to death from the Chinese Longitudinal Healthy Longevity Survey (CLHLS). The 6-month prevalence of toothache and jaw joint pain or facial pain for older adults in the last year of life to death were 14.1% and 4.5%, respectively. Older adults who had lower socioeconomic status, were smokers, and had any chronic disease tended to have orofacial pain symptoms. This study generated interesting but counterintutive findings that Chinese older adults who brusehed their teeth at least daily and those who had at least one natural teeth were more likely to have orofacial pain. It is important to include dental care as a part of end-of-life medical treatment.

